# Synergistic Effect of Adjustments of Elastic Stockings to Maintain Reduction in Leg Volume after Mechanical Lymph Drainage

**DOI:** 10.1155/2014/640189

**Published:** 2014-09-21

**Authors:** José Maria Pereira de Godoy, Renata Lopes Pinto, Ana Carolina Pereira de Godoy, Maria de Fátima Guerreiro Godoy

**Affiliations:** ^1^Department of Cardiology and Cardiovascular Surgery, Medicine School, FAMERP and CNPq (National Council for Research and Development), 15025120 São Jose do Rio Preto, SP, Brazil; ^2^Godoy Clinic, Avenida Constituição 1306, 15025120 São Jose do Rio Preto, SP, Brazil; ^3^FAMERP and Clinical Research Group of the Godoy Clinic, 15025120 São Jose do Rio Preto, SP, Brazil; ^4^Medicine School of ABC and Clinical Research Group of the Godoy Clinic, 15025120 São Jose do Rio Preto, SP, Brazil; ^5^Medicine School, FAMERP and Clinical Research Group of the Godoy Clinic, 15025120 São Jose do Rio Preto, SP, Brazil

## Abstract

The objective of the present study was to evaluate the effect of elastic compression stockings on volumetric variations of lymphedematous limbs between mechanical lymph drainage sessions. Eleven patients with Grade II leg lymphedema, regardless of etiology, were evaluated in a randomized clinical trial. The ages ranged from 47 to 83 years old with a mean of 62.4 years. Participants were submitted to mechanical lymph drainage (RAGodoy) associated with adjusted and unadjusted knee-high elastic compression stockings (20/30 Venosan). The effect of these stockings on the maintenance of volumetric reductions between sessions of lymph drainage was assessed. In all, 33 evaluations were carried out, 18 of patients using well-adjusted stockings and 15 with badly-adjusted stockings. The differences in volumes were significant (unpaired *t*-test; *P*-value < 0.0001). Adjusting the compression provided by elastic stockings according to the size of the leg has a synergistic effect in reducing volume during mechanical lymph drainage.

## 1. Introduction

Use of elastic stockings is one of the main clinical approaches to the treatment of venous edema and lymphedema [1, 2]. The pressures introduced by compression hosiery, muscle activity, the environmental temperature, and the integrity of joint mobility are factors that can interfere in reductions in edema and in maintaining the size of limbs [2–5].

Elastic stockings exert working and resting pressures at the interface between the stocking and the skin which decreases from the top of the thigh to the foot thus facilitating drainage of fluids even at rest. Muscular activity causes pulse wave-type variations in pressure, similar to what occurs in the venous system by muscle contraction [6]. The integrity of the muscle “pumps” is crucial to effectively generate pressure gradients during activity [5, 6].

The best option is to continuously use elastic stockings throughout the day, but when this is not possible, their utilization during part of the day can be considered beneficial [7]. Care should be taken not only to use the correct size of stocking for each patient but also to avoid creases and folds as this may cause a tourniquet effect inhibiting venous return thereby aggravating the edema [8].

Lymphedema is a chronic disease that requires constant care Where elastic stockings are indicated as important approach to treatment [9–11]. The effect of adjustments, which are necessary to maintain compression with the reductions in edema, on the functioning of these stockings has not been reported in the literature. The objective of the present study was to evaluate the effect of elastic compression stockings on volumetric variations of lymphedematous limbs between mechanical lymph drainage sessions.

## 2. Method

### 2.1. Study Design

Eleven patients with Grade II leg lymphedema, regardless of etiology, were evaluated in a randomized clinical trial. Participants were submitted to mechanical lymph drainage (RAGodoy) associated with adjusted and unadjusted knee-high elastic compression stockings (20/30 Venosan) used to maintain volumetric reductions between sessions.

### 2.2. Patients

Eleven female patients of the Clinica Godoy with ages ranging from 47 to 83 years old and with a mean of 62.4 years were enrolled in this study. The inclusion criteria were Grade II lymphedema of the legs regardless of etiology as evaluated by bioimpedance. Patients with infections and those with greatly reduced joint mobility and with arterial disease were excluded from the study.

### 2.3. Treatment

Participants were submitted to mechanical lymph drainage for two hours per day associated with well-adjusted or unadjusted elastic knee-high compression stockings (20/30 Venosan). The study participants were randomly divided into two groups by a simple raffle. Group I used elastic stockings for a week and then started mechanical lymph drainage sessions without adjustment of the stockings; Group II used elastic stockings from day 1 with the stockings being adjusted at each session. The study was carried out over five consecutive days with evaluations by bioelectrical impedance being carried out before and after the sessions. The patients were requested to use the stockings continuously (day and night).

The legs were measured after each session to check the size of the elastic stocking. Exchange of stockings in Group II was indicated when volumetric losses were observed in this case; the legs were measured again. InBody S10 body composition analyzer (BioSpace, Seoul, Korea), bioimpedance equipment was used; this device evaluates variations in the extremities and trunk and the intra- and extracellular mobilization of water.

The unpaired *t*-test was used for statistical analysis with an alpha error of 5% (*P* value < 0.05) being considered acceptable. The study was approved by the Research Ethics Committee of the Medicine School in São José do Rio Preto (FAMERP) number 172.293.

## 3. Result

**Figure 1:**
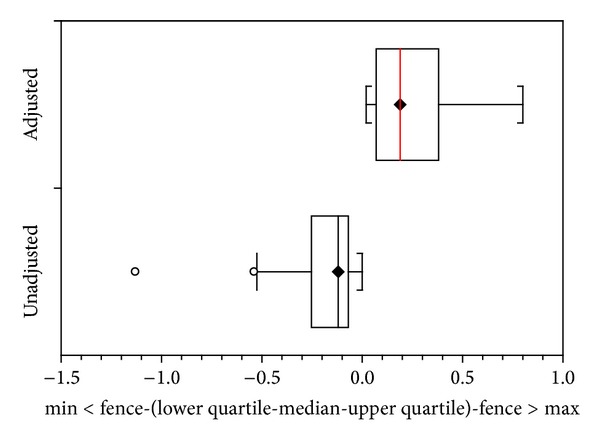
Box and whisker plot 1 shows changes in volume before and after treatment of lymphedema evaluated by bioimpedance in stockings adjusted and unadjusted.

In all, 33 evaluations were carried out, 18 of patients using well-adjusted stockings and 15 with unadjusted stockings (Table 1). The differences in volumes were significant, Figure 1, (unpaired *t*-test; *P* value < 0.0001).

## 4. Discussion

The present study shows the importance of adjusting elastic stockings to maintain the results obtained with lymph drainage. The losses of each session and the maintenance of the results between treatment sessions were evaluated. The correct size of stockings and their adjustment were important in maintaining the volumetric losses.

The idea of this study arose from daily observations that elastic stockings lose their effectiveness when the size of the limb reduces thus failing to maintain losses achieved during lymph drainage sessions. Reductions of around 200 to 300 mL make adjustment of the stockings necessary in order to maintain volumetric losses. However, there are no studies in the literature that report on this finding. It is also important to remember that creases, folds, and other mechanisms that might lead to a tourniquet effect must be avoided [8].

In the treatment of lymphedema, an association of therapies may have a synergistic effect to reduce the volumes of limbs. Lymph drainage, compression mechanisms, and exercising are the cornerstones of this therapy. Even so, it is essential to monitor the use of stockings throughout treatment not just prescribe them but to evaluate their efficacy. The loss of elasticity of stockings with volumetric reductions of the limb should be checked at each evaluation of these patients.

Lymph drainage in isolation reduces the amount of edema and can normalize the size of the limb in some cases; however, maintenance of results using an elastic stocking is necessary.

Nonelastic bandages are a first option in the treatment of lymphedema, but bandaging must be carried out by specialized professionals who are not always available. Thus, elastic stockings are an important alternative; to achieve the best results, stockings need to be checked regularly and the size adjusted when necessary [8, 12]. There are two possible manners to adjust the compression of these stockings: using a different size of elastic stocking or using two overlying stockings with this latter option reducing the operational costs.

A critical aspect of this study was the use of stockings at night which is questioned in venous disease and contraindicated in chronic arterial disease. Elastic compression stockings are manufactured for medical purposes and so the specialist must assess the indications and contraindications. Although lymphedema patients generally tolerate this type of treatment, they were advised to remove the stockings if they suffered pain or felt uncomfortable during the night.

Mechanical lymph drainage (RAGodoy) was used in this study; this technique uses muscle work, in this case associated with the elastic compression stocking. The use of elastic stockings with these devices is not always well tolerated and so for this reason a compression stocking with a 20/30 mmHg pressure gradient was chosen for this study. Inelastic bandages and low-stretch stockings are tolerated better.

## 5. Conclusion

Adjustments to elastic compression stockings according to the volume of leg cause a synergistic effect to further reduce size of the limb during mechanical lymph drainage.

## Figures and Tables

**Table 1:** Variations in bioimpedance of patients using unadjusted and well-adjusted elastic stockings during a 5-day program of mechanical lymph drainage.

Unadjusted Group 1	Adjusted Group 2
+0.38	−0.08
+0.3	−0.03
+0.17	−0.07
+0.02	−0.12
+0.24	−0.16
+0.43	−0.12
+0.8	−1.13
+0.55	−0.21
+0.04	−0.12
+0.17	−0.3
+0.22	−0.14
+0.11	−0.07
+0.19	0.0
+0.07	−0.54
+0.02	−0.29
	−0.24
	−0.07
	−0.04
